# Service use, unmet needs, and barriers to services among adolescents and young adults with autism spectrum disorder in Poland

**DOI:** 10.1186/s12913-019-4432-3

**Published:** 2019-08-20

**Authors:** Mateusz Płatos, Ewa Pisula

**Affiliations:** Faculty of Psychology, University of Warsaw, Stawki 5/7, 01-909 Warsaw, Poland

**Keywords:** Autism spectrum disorder, Service use, Adolescence, Young adulthood

## Abstract

**Background:**

Despite a growing number of adolescents and adults diagnosed with autism spectrum disorder (ASD), little is known about service needs and barriers to services in this population. Existing research shows that youth with ASD are more underserved as they approach final years of their high school education and that adequate services for individuals with ASD after transition to adulthood are even scarcer. However, few studies have directly compared differences in service availability between adolescents and adults with ASD, and even fewer studies are published on service use outside Anglo-Saxon countries. The purpose of the present study was to examine service access, perceived barriers, and unmet needs, as reported by parents of adolescents and young adults with ASD in Poland.

**Methods:**

The study used a subsample of parents of young people with ASD (aged 12–38 years; *N* = 311) from the Polish Autism Survey – a survey covering different areas of functioning of people with ASD in Poland, based on a convenience sample. Responding parents were recruited via different service providers, social media, and press, and completed a survey using a web platform or a paper-and-pencil questionnaire.

**Results:**

As expected, adults used services less often than adolescents, with 80.1% of adolescents and 61.1% of adults with ASD using services in the previous 12 months. Mental health services were among the most used and the most needed services, followed by educational services, while needs for sensory/motor services remained largely unmet. Young people with a coexisting intellectual disability used more services than those without it. Non-governmental organizations, private clinics, and schools were the most common service providers. Parents indicated that most of young people with ASD had unmet service needs for services (93.5%) and faced barriers to access them (82.7%). Low-income families and those living outside large cities were at the highest risk of facing barriers to service access.

**Conclusions:**

The results confirm still a thin body of evidence from different countries suggesting that adolescents and adults with ASD were both largely underserved populations. Policy-makers should address economic, regional, and age-related inequities in access to services for individuals with ASD.

## Background

There is a growing recognition that services for people with autism spectrum disorder (ASD) should accommodate the rising number of those who transition from school and enter adulthood [1, 2]. Adults with ASD need ongoing support to meet increasing social demands related to independent living, forming friendships and intimate relationships, and successful employment [3]. No more than about 20% of the adults with ASD are estimated to have good or very good outcomes in these areas [4, 5]. In turn, social isolation and unemployment likely contribute to alarmingly high rates of depression, anxiety, and other mental health problems [6, 7] and higher rates of suicidal ideation in this population [8]. Clearly, there is a need for improving quality and accessibility of services for adults and transitioning adolescents with ASD, but the empirical data to guide this process is scarce, especially outside the United States and Canada.

Various individual and social factors determine service use. According to the Behavioral Model of Health Services Use [9, 10], *predisposing factors* (such as age or sex) and *need factors* (such as health or disability status) affect the likelihood that a person will seek for services, whereas *enabling factors* (such as income or physical distance to service providers) enable or impede access to those services. In terms of this framework, age can be considered both a predisposing factor (if the need for services is a function of age) and enabling factor (if access to services is a function of age). However, existing studies on service use among people with ASD rarely cover both adolescents and adults, making it difficult to compare patterns of service use and needs across these developmental periods. Available studies from the United States and Canada suggest that outpatient, behavioral service use among people with ASD decreases from childhood to adulthood [11, 12] and continues to decrease towards late adulthood [13], while the use of medications and in-patient mental health services rises [11, 14]. Similarly to the U.S., in Poland, the end of high school is considered a crucial transitionary period and, unfortunately, also a moment when youth with ASD become ineligible for services, although their needs for services remain high [15]. According to an American study, two-fifths of young adults (aged 19–23) with ASD haven’t used any services after graduating from high school, compared to only 6.5% not using services before graduation [16]. Taken together, these data suggest low availability of adequate services for young adults with ASD. Further, the data on service use may be overestimated because most studies draw samples from single public (e.g. Medicaid in the U.S.; [11, 12]) or private [17] insurance records that store only data of people who have accessed services, or from special education enrollment databases [16]. Thus, individuals attending mainstream schools who do not use any services (or do not have insurance coverage) were unlikely to be included in those studies.

Other predisposing factors (e.g. gender), need factors (e.g. cognitive abilities and coexisting mental health problems) and enabling factors (e.g. income and area of residence) were rarely taken into consideration in the previous research [1]. Some research suggests that individuals from low-income households and racial/ethnic minorities are at higher risk of having unmet service needs [16, 18]. Little is known about other factors, such as the impact of a coexisting intellectual disability on access to services [13, 19]. Moreover, these individual risk factors are rarely considered in the examination of organizational and financing barriers for people with ASD and their families to using services (for exceptions: [13, 15]). Without an understanding of barriers to service access, research revealing low service participation has little application value.

Most of the available studies have been conducted in the United States, Canada, and other English-speaking countries. Although we found a small number of studies, concerning mostly children, conducted in Western Europe [20] and Southeastern Europe [21], to our knowledge, there is no study published in an international journal on services for adolescents and adults with ASD in Central and Eastern Europe. Therefore, little is known from previous studies about the patterns of service use and needs that can be generalized to other countries across various sociocultural, economic, and systemic factors.

Poland is one of the most populous and economically developed countries in Central and Eastern Europe. Actual data on autism prevalence in Poland is not available. The median prevalence of autism spectrum disorder in a general population, as reported in different countries since the year 2000, is estimated at 62/10000 [22]. Applying this estimate to the Polish population data [23] results in the approximate number of 13.5 thousand adolescents (12–17 years old) and 74 thousand of young adults (18–39 years old) on the autism spectrum. Similarly to other countries, the number of people who are diagnosed with ASD and receive services on that basis in Poland rises. For example, the number of students that received support under special education title because of autism or Asperger’s Syndrome doubled in just three years, from 2013 to 2016 [24]. This reflects a recent spurt in autism diagnoses in Poland, which may result from better availability of diagnostic measures [25] and higher public awareness of autism [26]. However, accessible data from educational [24] and health registers [27] do not cover adults with ASD who may be the most underdiagnosed and thus the most underserved population of people with ASD, resembling the situation in others countries [28].

Healthcare and education in Poland are predominantly public. Students can learn in the mainstream, integrated, or special classrooms until the age of 24, but people without an intellectual disability who attend high school typically end their secondary education at the age of 18–19. The National Health Fund, the Ministry of Health agency, finances both diagnostic and therapeutic services for people with ASD by contracting public, non-governmental, and private providers. Coverage of public health insurance is almost universal [29]. Public health services providers for people with ASD do not equally cover all the regions of Poland. In 2015, only a few, major cities (3% of all cities in Poland) had at least one service provider with a contract from the National Health Fund on the treatment of people with ASD [30]. Given the relatively large area of Poland, the distance may be an important barrier to services for people with ASD and their families. Lastly, there are several types of institutions, such as Community Self-Help Homes or Occupational Therapy Workshops, that organize social and vocational rehabilitation, as well as day and residential centers for adults with disabilities. In practice, most of these institutions are not available to people with ASD. Previous research points out to common barriers to the use of services by people with ASD, such as low staff-to-service-users ratio, no autism-related training of the staff or overcrowded rooms in residential centers [31]. Consequently, the public system of support for people with ASD is heavily supplemented by non-governmental organizations (run most often by parents) and a growing private sector. The former are co-funded by governmental agencies, most notably the State Fund for Rehabilitation of Disabled Persons, while the latter relies on out-of-pocket payments of people with ASD and their families.

The goal of the present study was to fill the gap in our understanding of service use, unmet needs for services, and barriers experienced by adolescents and adults with ASD in Poland. Moreover, the study aimed at identifying predisposing and need factors (analyzed jointly and hereafter referred to as ‘predisposing factors’) that impact service use, as well as enabling factors that contribute to unmet service needs and barriers to service use. ‘Services’ were understood as interventions aimed at improving the mental, social, and emotional well-being of individuals with ASD, including psychotherapeutic and instructional approaches. ‘Unmet needs’ were defined as services that people would like to use, but they do not have access to them. The three main research questions are outlined below.

1. What services do people with ASD use and what are predisposing factors to service use?
2. What are unmet needs for services and enabling factors that prevent service use?
3. What barriers to services do people with ASD face and who is at the greatest risk of facing those barriers?

## Methods

### Sample and procedure

The study is a secondary analysis of data retrieved from the Polish Autism Survey – a nationwide survey covering major areas of functioning of adolescents and adults with ASD in Poland [32] aged from 12 to 52. The data were collected from people with ASD and their parents in 2014. The present analyses are based on the data from parents who reported on service use of their adolescents and young adults with ASD (*N* = 311). The survey data were included in the study if the following criteria were satisfied: (a) a responding parent completed all the sections of the survey relevant to this study, (b) a young person, whose parent filled out the survey, had a diagnosis of childhood autism, atypical autism, Asperger’s Syndrome, or other pervasive developmental disorders, according to International Classification of Diseases, Version 10 [33], (c) and was 12–39 years old.

For the purpose of participants’ recruitment in the original study, service providers that offer therapeutic services for people with ASD in Poland were contacted via e-mail and telephone. Because of the lack of data on the demographic structure of this population in Poland, only convenience sampling could be used. Nevertheless, the effort was made to maximize the opportunity for all the potential participants to take part in the study. This was done by identifying and contacting all the institutions that offered support for individuals with ASD in Poland (*N* = 854): non-governmental organizations and clinics for people with autism (*n* = 254), mental health clinics (*n* = 42), day hospitals (*n* = 43), educational counseling centers (*n* = 267) and occupational therapy workshops (*n* = 150). Schools with integration or special classrooms that were contacted (*n* = 98) had been randomly selected from the database stratified by region and size of the area of residence. In sum, 204 service providers responded to the request for collaboration and helped in the recruitment process (23.9% response rate). Moreover, an extensive campaign was carried out in the social media and the press to reach parents of people with ASD who did not use any services.

Individuals willing to take part in the study could fill out the survey online or use the paper-and-pencil version and send it back via attached, prepaid envelope. Most of the participants (92.9%) used the web-based version of the survey. All participants were assured about the confidentiality of the gathered data. For both online and paper-and-pencil versions of the survey, informed consent was obtained from the participants. The study received approval from the Ethical Board at the Faculty of Psychology, University of Warsaw.

The study participants were mostly mothers (87.8%; 10.3% fathers, 1.6% other caregivers or family members) of young people with ASD. Responding parents were predominantly middle-aged (49.5% were 40–49 years old) and most had a university (54.0%) or high school (36.3%) degree. Young people with ASD, whose parents responded to the survey, were from 12 to 38 years old (*M* = 16.57; *SD* = 5.15) and predominantly males (79.1%), with the female-to-male ratio of 1:3.8 being close to the ratios previously reported in this population [34, 35]. Almost half of the young people (45.3%) had a coexisting intellectual disability. Over half of the young people (58.2%) had a diagnosis of a coexisting mental health problem, similarly to international reports on that population [36, 37]. Participants lived in all the 16 Voivodeships (major administrative regions) of Poland and all types of residential areas with the biggest proportion of participants from large cities (35.7%) and Masovian Voivodeship, the most populous region in Poland (21.6%). Detailed demographic information is provided in Table 1.

**Table 1:** Demographic characteristics of the respondents

Characteristic	N	%
Young person characteristics		
Gender		
Male	246	79.1
Female	65	20.9
Age (years)		
12–14	147	47.3
15–17	69	22.2
18–24	73	23.5
25–39	22	7.1
Education		
Primary school	117	37.6
Middle school	82	26.4
High school	50	16.1
University	8	2.6
Completed education		
Less than high school	22	7.1
High school	15	4.8
University	3	1.0
Not responded	14	4.5
Diagnosis		
Childhood autism	132	42.2
Atypical autism	38	12.2
Asperger Syndrome	120	40.2
Other pervasive developmental disorders	11	3.5
Not specified	1.6	1.6
Comorbid intellectual disability		
Yes	141	45.3
No	170	54.7
Comorbid psychiatric diagnosis		
Yes	181	58.2
No	126	40.5
Household characteristics		
Household income (PLN)		
< 1501	64	20.6
1501–2500	61	19.6
2501–3500	55	17.7
3501–5500	54	17.4
> 5501	39	12.6
Not reported	38	12.2
Residential area		
Large city (> 500 k inhabitants)	111	35.7
Medium city (50-200 k inhabitants)	63	20.3
Small city (< 50 k inhabitants)	60	19.3
Village	73	23.5
Not reported	4	1.2

## Measures

The Polish Autism Survey was a nationwide study aimed at learning about the experiences and needs of adolescents and adults with ASD in Poland. The survey covered eight areas (demographic information and living arrangements, health, education, peer relationships, leisure and interests, adaptive skills, service use, work and vocational support) of which this paper focuses on service use. A detailed description of the survey development can be found in Płatos and colleagues [32].

### Demographic information

Demographic measures included questions concerning both the young person’s with ASD and the responding parent’s age, gender, education, area of residence, household income, type of ASD diagnosis, coexisting mental health problems and an intellectual disability. Due to the prevailing monoethnic structure of the Polish society, an ethnic/racial identity was not asked [38].

### Met and unmet service needs

To learn about people’s with ASD met and unmet service needs, we provided a list of 11 types of services (as listed in Table 3) and an open-ended question to complement the list with self-indicated types of services. For met service needs, parents were asked to choose all the services their child used in the past 12 months. For unmet service needs, parents were asked to indicate all the services they would like their child to receive, which they weren’t receiving at the time of the study. The provided list of services included therapeutic services related to ASD or common co-occurring problems. Categories of services were generic, in recognition of the fact that most agencies in Poland take an eclectic approach to treatment and people often cannot identify a specific method that is used (e.g. Applied Behavior Analysis, Cognitive-Behavioral Therapy). Additionally, people were asked how many service hours they received, where the services were provided, and service funding (i.e. are they self-paid or publicly funded).

### Barriers to access services

Participants were asked if there was anything that makes it difficult for them to access therapeutic services. Respondents were provided with a multiple choice list of five potential barriers (as listed in Table 4) but could also indicate other barriers in a blank field.

### Statistical analysis

For the current analyses, services were grouped into mental health services (including individual therapy, group therapy, and psychological/psychiatric consultations) and sensory/motor services (including sensory integration training, auditory integration training, animal-assisted therapy, biofeedback, art therapy and physical therapy), while educational services and speech and language therapy (SLT) were analyzed separately. Poisson regression and negative binomial regression with log link were performed for predicting the number of services used or needed by young people with ASD and logistic regression was used to predict the likelihood of using a single type of service. Stepwise logistic regression analysis was performed to determine which demographic and socioeconomic factors increased the risk of each of the five barriers to service use. IBM SPSS 24.0 software was used for the analysis.

## Results

### What services do people with ASD use and what are predisposing factors to service use?

Table 2 presents the characteristics of services used by young people with ASD. Seventy-four percent (74.3%) used at least one therapeutic service in the 12 months preceding the study. Services were provided mostly in non-public (private or non-profit) therapeutic centers, followed by public mental health or autism clinics, schools, and educational counseling centers. The mean number of services used by young people was 2.7 (range from 0 to 9; *SD* = 2.3) and the median number of hours of services per week was 1–3 h.

**Table 2:** Characteristics of services received by young people with ASD

Service characteristics	N	%
*Services received per week* (*N* = 305)		
< 1 h	30	9.8
1–3 h	94	30.8
4–10 h	77	25.2
11–18 h	13	4.3
> 19 h	11	3.6
none	80	26.2
*Funding of services* (*N* = 223)		
Public	93	41.7
Out-of-pocket	46	20.6
Mixed	84	37.7
*Where the services were provided* (*N* = 231)		
Public mental health clinic^a^	70	30.3
Private or NGO-based clinic	127	55.0
School	106	45.9
Educational counseling centers	51	22.1
Therapeutic camp	52	22.5
Home-based	41	17.7
Other^b^	27	11.7

NGO = Non-governmental organization

^a^ Including Clinics for People with Autism; ^b^ Includes combined categories: “Occupational Therapy Workshop”, “Community Self-Help Home”, “Day hospital”, and “Other”

As shown in Table 3, educational services were used most often (42.4%) followed by psychiatric or psychological consultations (40.2%), group therapy (33.4%), speech and language therapy (SLT) and individual therapy (30.9%).

**Table 3:** Services used and needed by individuals with ASD

Type of service	Service use (*N* = 311)		Unmet service needs (*N* = 306)	
	N	%	N	%
*Mental health services*				
Psychiatric/psychological consultations	**125**	**40.2**	77	25.2
Individual therapy (incl. Psychotherapy, ABA, other approaches)	96	30.9	**135**	**44.1**
Group therapy (incl. Social skills training, support group)	**104**	**33.4**	**155**	**50.7**
*Sensory/motor services*				
Sensory Integration Therapy	80	25.7	86	28.8
Auditory Integration Training	18	5.8	65	21.2
Art therapy	45	14.5	**92**	**30.1**
Physical therapy	26	8.4	64	20.9
Biofeedback	31	10	69	22.5
Animal-assisted interventions	50	16.1	83	27.1
Educational services	**132**	**42.4**	91	29.7
Speech and language therapy	103	33.1	57	18.6
Other	18	5.8	11	3.6
None	80	25.7	20	6.5

Note. Three most frequently indicated services and unmet needs were bolded: ABA = Applied Behavior Analysis

Negative binomial regression with log link was performed to predict the total number of services used by young people with ASD, based on their sex, age, autism diagnosis subtype (childhood autism vs. other diagnoses), coexisting mental health problems, and a coexisting intellectual disability. The model was statistically significant (*χ*^*2*^(5) = 46.1, *p* < .001). Young people with ASD and a coexisting intellectual disability used 1.562 (95% CI, 1.239 to 1.971) times more services than those without an intellectual disability (*p* < .001). For every extra year of age, young people with ASD used .932 (95% CI, .911 to .954) times fewer services (*p* < .001). All the other predictors were not statistically significant.

Based on the same set of factors, two Poisson regression analyses were performed to predict the number of mental health services and sensory/motor services used by young people with ASD. The model for mental health services was statistically significant (*χ*^*2*^(5) = 16.5, *p* = .006). Young people with ASD and coexisting mental health problems used 1.302 (95% CI, 1.021 to 1.660) times more mental health services than those without mental health problems (*p* = .033). For every extra year of age, young people with ASD used .962 (95% CI, .938 to .987) times less mental health services (*p* = .003). All the other predictors were not statistically significant.

The model for sensory/motor services was also statistically significant (*χ*^*2*^(5) = 90.2, *p* < .001). Young people with ASD and a coexisting intellectual disability used 2.491 (95% CI, 1.839 to 3.374) times more sensory/motor services than those without an intellectual disability (*p* < .001). For every extra year of age, young people with ASD used .910 (95% CI, .878 to .943) times fewer services (*p <* .001). Those with coexisting mental health problems used .769 (95% CI, .588 to 1.004) fewer sensory/motor services than those without, although this result was significant only on a trend level (*p* = .054). All the other predictors were not statistically significant.

Logistic regression was conducted to identify predisposing factors for using educational services by young people with ASD, based on the same set of predictors as in the previous analyses. The model was statistically significant (*χ*^*2*^(5) = 47.0, *p* < .001). The likelihood of using educational services decreased with age, with the odds .807 (95% CI, .748 to .871) smaller with every extra year (*p* < .001). All the other predictors were not statistically significant.

Another logistic regression was performed to predict the likelihood of SLT. The regression model was statistically significant (*χ*^*2*^(5) = 81.0, *p* < .001). Gender was a significant predictor of using SLT (*p* = .044) with females being 1.984 (95% CI, 1.018 to 3.867) more likely to use it than males. Young people with ASD and a coexisting intellectual disability had 3.053 (95% CI, 1.628 to 5.727) greater odds of using SLT than those without it (*p* = .001). Similarly, young people with a diagnosis of childhood autism had 2.334 (95% CI, 1.227 to 4.437) greater odds of using SLT than people with a diagnosis of other autism subtypes (*p* = .01). The likelihood of using SLT decreased with age, with the odds .790 (95%, .723 to .864) smaller with every extra year (*p* < .001). Having coexisting mental health problems was not a significant predictor in this model (*p* = .808).

### What are unmet needs for services and enabling factors that prevent service use?

Respondents were asked what services they would like their children to receive that they did not use at the time of the study. As much as 93.5% of parents indicated at least one service. A mean number of services needed was 3.2 (range 0–11, *SD* = 2.3). Parents most often indicated that young people with ASD needed group therapy (50.7%), individual therapy (44.1%), and art therapy (30.1%; see Table 3).

A series of regression analyses was conducted to identify enabling factors that predict unmet service needs among young people with ASD. Negative binomial regression with log link was used to ascertain the effects of income, residential area, young person’s education, maternal education, as well as sex and age on the overall number of unmet service needs. The model was not statistically significant (*χ*^*2*^(11) = 15.2, *p* = .173). The same set of factors was used in Poisson regression analyses to predict the number of mental health services and sensory/motor services. The model for mental health services did not reach significance (*χ*^*2*^(11) = 12.4, *p* = .335), whereas the model for sensory/motor services was statistically significant (*χ*^*2*^(11) = 25.5, *p* = .008). People with higher income had .929 (95% CI, .874 to .988) fewer unmet needs for sensory/motor services per every category of income (*p* = .018; see Table 1). Moreover, women with ASD had 1.227 (95% CI, 1.007 to 1.619) more unmet needs for this type of services than men with ASD (*p* = .044). All the other predictors were not statistically significant. Finally, two logistic regression models to predict the likelihood of having unmet needs for using educational services and SLT, with the same set of factors as in previous analyses, were not statistically significant (*χ*^*2*^(11) = 14.8, *p* = .192 and *χ*^*2*^(11) = 13.5, *p* = .260, respectively).

### What barriers to services do people with ASD face and who is at the greatest risk of facing those barriers?

Eighty-two percent (82.7%) of the parents reported at least one barrier to service use (see Table 4). The mean number of barriers was 1.6 (range 0–6; *SD* = 1.22).

**Table 4:** Barriers to service use reported by parents of young people with ASD (*N* = 307)

Type of barrier	N	%
Services are hardly available	132	43.0
I/My child doesn’t qualify for admission to services	49	16.0
Cost of services is too high	116	37.8
Information about services are hard to get	64	20.8
It’s difficult to get to the place where services are offered	85	27.7
Other	43	14.0
None of the above – I have a suitable access to therapeutic services	53	17.3

The two most frequently reported barriers to services were the unavailability of the desired service (43.0%) and the high cost of services (37.8%). Each of the other predefined barriers was chosen by at least 15% of the respondents (see Table 4).

Fourteen percent (14.0%) indicated other barriers to services. The most common response concerned the lack of time for attending a therapy (4.6%), due to young person’s or parents’ heavy workload, or conflicting hours of therapy and school. The second most prevalent response included a perceived lack of motivation among young people to attend therapy (3.6%) or open resistance to do so. Most of the remaining answers were reiterating the barriers listed in the original question, most commonly a long distance to the place where services were provided.

As revealed by stepwise logistic regression analyses, several significant factors increased the risk of facing barriers to services (see Table 5).

**Table 5:** Odds ratios (95% CI in parentheses) for factors associated with barriers to services for young people with ASD (parents’ reports)

Factor	Services not available	Don’t qualify to services	Services too costly	No info on services	Services too far
Being younger (12–14 y.o.)^a^			2.16 (1.28–3.63)		
Lower income^b^			1.24 (1.05–1.46)		
Attending integrated class^c^			2.02 (1.13–3.62)		
Living in a medium city^d^	3.07 (1.52–6.19)				
Living in a small city^d^					2.52 (1.24–5.11)
Living in a village^d^					4.07 (2.14–7.71)

Note. Analyses were performed separately for each barrier. Only significant factors (*p* < .05) are shown

^a^ 18–24 years old range as a reference. ^b^ Change in odds with every decrease of the income level. ^c^ Attending a special education classroom as a reference. ^d^ A large city as a reference

The youngest age group of the sample (12–14 years old) was found to be associated with parents reporting that services were too costly. The likelihood of facing the cost barrier was also increased by low household income and attending an integrated (vs. special) classroom by young people with ASD. Finally, various risks were associated with living in a village, as well as a medium and small cities, compared to large cities. Living in a medium city increased the odds of reporting that services are unavailable. Both living in a small city and in a village increased the odds of reporting that services were too far away.

Ineligibility to services and not having information on services was not predicted by any significant factors. Furthermore, gender, autism diagnostic category, a coexisting intellectual disability, and currently attended education level were not significantly associated with an increased likelihood of facing any of the service barriers.

## Discussion

The present study confirmed that adolescents and young adults with ASD in Poland are largely underserved populations, with 25.7% (19.9% adolescents and 38.9% young adults) not using any therapeutic services in the previous 12 months. Low service utilization, especially among young adults with ASD, corroborates research findings from other countries, for example, 39.1% of young adults with ASD in the U.S. did not use any services in prior 2 years [16]. However, rates of service use should be interpreted with a reference to service needs. Parents of both adolescents and young adults almost universally (93.5%) indicated that their children did not receive all the services they needed. Critically, about one-fourth (24.5%) of parents declared that young people with ASD have not used any services in a prior year though needed them. Therefore, one in four young people with ASD can be considered excluded from the support system. Given the convenience sampling method used in this study and service providers being an important source of participants, this rate is probably still underestimated.

Consistent with the data from the U.S. and Canada [12, 13], mental health services were the most used and still the most needed services. Types of services in this category included both treatment of core problems associated with ASD (e.g. Applied Behavior Analysis, Social Skills Training) and psychotherapeutic approaches for the treatment of coexisting mental health problems. High level of needs for the latter type of services is not surprising, given very high rates of coexisting mental health problems in the sample. Young people with ASD and coexisting mental health problems used 30% more mental health services than those without these problems. Elevated incidence of anxiety and mood disorders, as well as conduct disorders and attention deficit hyperactivity disorder, is reported internationally in both adolescent and adult populations of people with ASD [36, 37]. Findings from the current study indicate that individual and group psychotherapy and, to the lesser degree, psychological and psychiatric consultations are largely underserved in young people with ASD.

Another result puts this finding in the perspective – only 30.3% of young people with ASD used services provided by public mental health clinics that are main institutions offering state-funded mental health support. In contrast, 55.0% of young people with ASD used private or NGO-based services (the latter can operate commercially or using state funds). In Poland, mental healthcare is largely underfunded, with only 8.5 psychiatrists per 100,000 people, compared to the average 17.2 per 100,000 in the European Union [29]. Long waiting time for psychiatric and psychological services funded by the National Health Fund results in the proliferation of private mental health services. In the present sample, 20.6% of young people with ASD used only out-of-pocket services, while 37.7% combined them with state-funded services.

Mental health services include also treatment approaches for the core deficits in ASD, most notably Applied Behavior Analysis therapy and Social Skills Trainings for teaching communication and social skills to people with ASD. There are several evidence-based Social Skills Trainings for this population [39], but none of them is available in Poland. Moreover, Clinics for People with Autism, which are specialized facilities that often provide Social Skills Trainings and Applied Behavior Analysis treatment, are scarcely and unevenly distributed in Poland [30], again directing many people with ASD to out-of-pocket services. Taken together, these results emphasize the critical importance of increasing accessibility of the psychological and psychiatric treatment of both core and coexisting problems that affect adolescents and adults with ASD.

Educational services, SLT, and Sensory Integration Training were each used by more than 25% of young people with ASD. Sensory Integration Training and other sensory/motor services were also highly demanded – in all six types of treatments included in this category, unmet service needs exceeded service use (e.g. 30.1% demanded Art therapy and only 14.5% actually received it). In Poland, these services are provided mostly in education settings (i.e. schools and educational counseling centers) and by private therapeutic centers. This results in limited access to sensory/motor services and SLT for a few groups of people with ASD. First, in the present study, people with lower income had a greater number of unmet needs specifically for sensory/motor services. Second, parents whose children attended integration classes, compared to special classes, were at higher risk of facing too high costs of services. Third, many adults are no longer eligible for services in education settings, so they must rely on out-of-pocket services. In contrast to school and educational counseling centers that were service providers for, respectively, 45.9 and 22.1% of young people with ASD, institutions that provide support to adults with ASD, such as Occupational Therapy Workshops or Community Self-Help Homes almost did not appear in the results of the survey. This result corroborates a previous Polish report that pointed out that these institutions are mostly unavailable for people with ASD [31].

The aim of the study was also to identify predisposing and enabling factors determining access to services. Age was the most consistent predisposing factor. Both overall use of services and use of each type of services decreased with age. However, age was not an enabling factor. In contrast to one American study [12], our study did not show an increase in unmet service needs with age. This suggests that an age-related decrease in service use among people with ASD may reflect fewer needs for services in older age groups. Alternatively, it can be a cohort effect, with an older cohort being less aware of available services and thus indicating fewer unmet needs.

Another important predisposing factor was related to an intellectual disability. Sensory/motor services and SLT were more often used by individuals with a coexisting intellectual disability than without it. Increased use of these services indicates two areas of difficulties that might be more pronounced among individuals with a coexisting intellectual disability – language development and sensory sensitivity. It is important to note that although the use of these services generally decreases with age, they remain important for individuals with a coexisting intellectual disability, so they should be sustained also beyond the education settings.

The present study explored the reasons why individuals with ASD could not access needed services. Service barriers outlined by Shattuck and colleagues [1] included availability, affordability, accessibility, acceptability, and accommodation. In the light of the present study, problems with availability (services don’t exist) and affordability (services are too costly) were most prominent, with unique barriers faced by different subpopulations of people with ASD.

For 43.0% of participants, services were hardly available – this highlights the fact that services for adolescents and adults may simply not exist in many regions of Poland, which is corroborated by other reports [30, 31]. As mentioned, specialized institutions for adults with ASD, such as Clinics for People with Autism and Occupational Therapy Workshops, were rarely used by participants in our and other [31] studies. Moreover, the area of residence was an important enabling factor for young people with ASD. More than a quarter of parents indicated services were too far away from their home. Participants from medium cities (i.e. having 50–200 thousand inhabitants) were at 3 times higher risk of finding services unavailable, compared to those living in large cities. This resembles the broader context of specialized, ambulatory healthcare in Poland, which is mostly located in large cities and, as a result, less used by inhabitants of smaller cities and rural regions [40]. There are 70 cities of medium size in Poland and most of them do not have any services for individuals with ASD funded by the National Health Fund [30].

The second most pronounced barrier was related to the affordability of services. Although in Poland both healthcare and education are predominantly public, for the reasons outlined earlier in this discussion, many services are available only commercially. Predictably, low-income parents were at higher risk of facing a financial barrier, as well as parents of young people attending integrated classes that provide fewer services than special classes. The youngest people with ASD in the study (12–14 years old) were at highest risk of finding services too expensive. This result is consistent with the finding that the youngest people with ASD in our sample used the greatest number of services. Therefore, financial barriers may stem from the accumulated costs of education and other services that parents must cover.

Other barriers to services should be addressed. While “acceptability” of services was not specifically examined as a barrier, it was identified by many participants who wrote that they (or their children) did not like the services or were too burdened with school or work to participate. The existence of these types of barriers is consistent with a previous American research [13] and should be taken into account in designing services for people with ASD.

### Limitations

The study has several limitations. First, although we do not have data on the demographic characteristic of the population of young people with ASD and their parents in Poland, we can suspect that the sample may have overrepresented parents with higher education and living in large cities. Convenience sampling limits generalizability of the results. Thus, descriptive data should be interpreted with caution and reported demographic factors that influence service use should be taken into account. Second, as service providers were one of the distributors of survey questionnaires, it is likely that the number of people with ASD who did not use any services was underestimated. We diminished partially this limitation by carrying out a campaign in social and traditional media. Third, it was a self-report study, largely based on the internet survey, so we were unable to corroborate the data (including diagnosis) with other sources. Finally, young people with ASD who live independently or in the residential centers may have not been represented, as we relied on parents as informants.

The study also presents remarkable strengths that, we believe, outweigh its limitations. The samples sizes were relatively large that allowed to account for various factors influencing service use and needs. It is only one of the few studies on adolescents and adults that combine an examination of service use, unmet needs, and barriers. Finally, this is one of the first studies of its kind in Central and Eastern Europe, largely confirming that findings from studies conducted in English-speaking countries are generalizable, as well as pointing to the specific context of Polish institutions and services for people with ASD.

## Conclusion

This is the first, to our knowledge, internationally reported survey on service use, needs, and barriers experienced by adolescents and adults with ASD in Poland. Although some data indicate that the majority of these two populations may still lack a proper diagnosis, those who are diagnosed do not have sufficient access to the mental health services, such as individual therapy, social skills training, and psychiatric/psychological consultations, as well as sensory/motor services. The study also revealed economic and regional inequities in access to services, with people living in villages and small-to-medium cities finding services unavailable or too distant. In the next few years, the number of diagnosed adolescents and adults with ASD is expected to increase. Therefore, the results from our study emphasize a critical need for improving the availability, affordability, and acceptability of services for these populations.

## Data Availability

The datasets analyzed during the current study are not publicly available due to data confidentiality but are available from the corresponding author on reasonable request.

## Abbreviations

ABA: Applied Behavior Analysis
ASD: Autism spectrum disorder
NGO: Non-governmental organization
SLT: Speech and language therapy

## References

[1] Shattuck PT, Roux AM, Hudson LE, Taylor JL, Maenner MJ, Trani J-F (2012). Services for Adults with an autism Spectrum disorder. Can J Psychiatr [Internet]..

[2] Lake JK, Perry A, Lunsky Y (2014). Mental health Services for Individuals with high functioning autism Spectrum disorder. Autism Res Treat [Internet]..

[3] Howlin P, Moss P (2012). Adults with autism spectrum disorders. Can J Psychiatr.

[4] Eaves LC, Ho HH (2008). Young adult outcome of autism spectrum disorders. J Autism Dev Disord.

[5] Howlin P, Moss P, Savage S, Rutter M (2013). Social outcomes in mid- to later adulthood among individuals diagnosed with autism and average nonverbal IQ as children. J Am Acad Child Adolesc Psychiatry.

[6] Lugnegård T, Hallerbäck MU, Gillberg C (2011). Psychiatric comorbidity in young adults with a clinical diagnosis of Asperger syndrome. Res Dev Disabil.

[7] Hofvander B, Delorme R, Chaste P, Nydén A, Wentz E, Ståhlberg O (2009). Psychiatric and psychosocial problems in adults with normal-intelligence autism spectrum disorders. BMC Psychiatry.

[8] Cassidy S, Bradley P, Robinson J, Allison C, McHugh M, Baron-Cohen S (2014). Suicidal ideation and suicide plans or attempts in adults with asperger’s syndrome attending a specialist diagnostic clinic: a clinical cohort study. Lancet Psychiatry.

[9] Andersen RM (1995). Revisiting the behavioral model and access to medical care: does it matter?. J Health Soc Behav.

[10] Babitsch B, Gohl D, von Lengerke T. Re-revisiting Andersen’s behavioral model of health services use: a systematic review of studies from 1998–2011. GMS Psycho-Social-Medicine. 2012 Jun;9. 10.3205/psm000089 PMC3488807 23133505

[11] Cidav Z, Lawer L, Marcus SC, Mandell DS (2013). Age-related variation in health service use and associated expenditures among children with autism. J Autism Dev Disord.

[12] Turcotte P, Mathew M, Shea LL, Brusilovskiy E, Nonnemacher SL (2016). Service needs across the lifespan for individuals with autism. J Autism Dev Disord.

[13] Vogan V, Lake JK, Tint A, Weiss JA, Lunsky Y (2017). Tracking health care service use and the experiences of adults with autism spectrum disorder without intellectual disability: a longitudinal study of service rates, barriers and satisfaction. Disabil Health J.

[14] Esbensen AJ, Greenberg JS, Seltzer MM, Aman MG (2009). A longitudinal investigation of psychotropic and non-psychotropic medication use among adolescents and adults with autism Spectrum disorders. J Autism Dev Disord.

[15] Taylor JL, Henninger NA (2015). Frequency and correlates of service access among youth with autism transitioning to adulthood. J Autism Dev Disord.

[16] Shattuck PT, Wagner M, Narendorf S, Sterzing P, Hensley M. Post–High School Service Use Among Young Adults With an Autism Spectrum Disorder. 2011;165(2):141–146. 10.1001/archpediatrics.2010.279 PMC3097532 21300654

[17] Ballard J, Crane DR, Harper JM, Fawcett D, Sandberg J (2016). Mental health service utilization in autism spectrum disorders. Res Autism Spectr Disord.

[18] Liptak GS, Benzoni LB, Mruzek DW, Nolan KW, Thingvoll MA, Wade CM (2008). Disparities in diagnosis and access to health services for children with autism: data from the national survey of children’s health. J Dev Behav Pediatr.

[19] Eklund H, Findon J, Cadman T, Hayward H, Murphy D, Asherson P (2018). Needs of adolescents and young adults with neurodevelopmental disorders: comparisons of young people and parent perspectives. J Autism Dev Disord.

[20] Rattaz C, Ledesert B, Masson O, Ouss L, Ropers G, Baghdadli A (2014). Special education and care services for children, adolescents, and adults with autism spectrum disorders in France: families’ opinion and satisfaction. Autism..

[21] Daniels AM, Como A, Hergüner S, Kostadinova K, Stosic J, Shih A (2017). Autism in Southeast Europe: a survey of caregivers of children with autism Spectrum disorders. J Autism Dev Disord.

[22] Elsabbagh M, Divan G, Koh YJ, Kim YS, Kauchali S, Marcín C (2012). Global prevalence of autism and other pervasive developmental disorders. Autism Res.

[23] Statistics Poland. Table 4. Population by sex and age in 2017. 2017. https://stat.gov.pl/download/gfx/portalinformacyjny/pl/defaultaktualnosci/5468/6/22/1/ludnosc_stan_i_struktura_30062017.zip. Accessed 10 May 2018.

[24] Ministry of National Education. Uczniowie posiadający orzeczenie o potrzebie kształcenia specjalnego w podziale na typy szkół oraz województwa w roku szkolnym 2016/2017. 2017. https://danepubliczne.gov.pl/dataset/dane_statystyczne_uczniow_z_orzeczeniem_o_potrzebie_ksztalcenia_specjalnego. Accessed 10 May 2018.

[25] Chojnicka I, Pisula E (2017). Adaptation and validation of the ADOS-2 , polish version. Front Psychology.

[26] Omyła-Rudzka M. Społeczny obraz autyzmu. Komun z badań. CBOS. 2015;47.

[27] Piskorz-Ogórek K, Ogórek S, Cieślińska A, Kostyra E (2015). Autism in Poland in comparison to other countries. Polish Ann Med.

[28] Brugha T, Mcmanus S, Bankart J, Scott FJ, Purdon S, Smith J (2011). Epidemiology of autism Spectrum disorders in adults in the Community in England. Arch Gen Psychiatry.

[29] Sowada C, Sagan A, Kowalska-Bobko I, Badora-Musiał K, Bochenek T, Domagała A (2019). Poland: health system review 2019. Health Syst Transit.

[30] Szymańska P (2016). Dziecko z autyzmem. Dostęp do diagnozy, terapii i edukacji w Polsce.

[31] Jankowska M, Rymsza A, Wildner E, Wroniszewska M, Kuruliszwili S, Raport KA (2013). Autyzm – Sytuacja Dorosłych.

[32] Płatos M, Gocłowska K, Wojaczek K, Woźniak-Rekucka P, Zawisny A, Pisula E, Płatos M (2016). Sytuacja młodzieży i dorosłych z autyzmem w świetle sondażu ogólnopolskiego. Ogólnopolski Spis Autyzmu Sytuacja młodzieży i dorosłych z autyzmem w Polsce. Warszawa: Stowarzyszenie Innowacji Społecznych ‘Mary i Max.

[33] World Health Organization (1992). The ICD-10 classification of mental and Behavioural disorders: clinical descriptions and diagnostic guidelines.

[34] Baio J, Wiggins L, Christensen DL, Maenner MJ, Daniels J, Warren Z (2018). Prevalence of autism Spectrum disorder among children aged 8 years - autism and developmental disabilities monitoring network, 11 sites, United States, 2014. MMWR Surveill Summ.

[35] Baxter AJ, Brugha TS, Erskine HE, Scheurer RW, Vos T, Scott JG (2015). The epidemiology and global burden of autism spectrum disorders. Psychol Med.

[36] Buck TR, Viskochil J, Farley M, Coon H, McMahon WM, Morgan J (2014). Psychiatric comorbidity and medication use in adults with autism Spectrum disorder. J Autism Dev Disord.

[37] Mattila M-L, Hurtig T, Haapsamo H, Jussila K, Kuusikko-Gauffin S, Kielinen M (2010). Comorbid psychiatric disorders associated with Asperger syndrome/high-functioning autism: a community- and clinic-based study. J Autism Dev Disord.

[38] Statistics Poland. Ludność. Stan i struktura demograficzno-społeczna - NSP 2011. 2017. http://stat.gov.pl/spisy-powszechne/nsp-2011/nsp-2011-wyniki/ludnosc-stan-i-struktura-demograficzno-spoleczna-nsp-2011,16,1.html. Accessed 10 May 2018.

[39] Wolstencroft J, Robinson L, Srinivasan R, Kerry E, Mandy W, Skuse D (2018). A systematic review of group social skills interventions, and meta-analysis of outcomes, for children with high functioning ASD. J Autism Dev Disord.

[40] Central Statistical Office (2017). Health and health care in 2016.

